# Enhancing single-cell encapsulation in droplet microfluidics with fine-tunable on-chip sample enrichment

**DOI:** 10.1038/s41378-023-00631-y

**Published:** 2024-01-02

**Authors:** Tao Tang, Hao Zhao, Shaofei Shen, Like Yang, Chwee Teck Lim

**Affiliations:** 1 Department of Biomedical Engineering, National University of Singapore, 117583 Singapore, Singapore; 2 Integrative Sciences and Engineering Programme, NUS Graduate School, National University of Singapore, 119077 Singapore, Singapore; 3 Shanxi Key Lab for Modernization of TCVM, College of Life Science, Shanxi Agricultural University, Taigu, Shanxi 030801 China; 4 Institute for Health Innovation & Technology, National University of Singapore, 117599 Singapore, Singapore; 5 Mechanobiology Institute, National University of Singapore, 117411 Singapore, Singapore; 6 Institute for Digital Molecular Analytics and Science, Nanyang Technological University, 636921 Singapore, Singapore

**Keywords:** Engineering, Chemistry

## Abstract

Single-cell encapsulation in droplet microfluidics is commonly hindered by the tradeoff between cell suspension density and on-chip focusing performance. In this study, we introduce a novel droplet microfluidic chip to overcome this challenge. The chip comprises a double spiral focusing unit, a flow resistance-based sample enrichment module with fine-tunable outlets, and a crossflow droplet generation unit. Utilizing a low-density cell/bead suspension (2 × 10^6^ objects/mL), cells/beads are focused into a near-equidistant linear arrangement within the double spiral microchannel. The excess water phase is diverted while cells/beads remain focused and sequentially encapsulated in individual droplets. Focusing performance was assessed through numerical simulations and experiments at three flow rates (40, 60, 80 μL/min), demonstrating successful focusing at 40 and 80 μL/min for beads and cells, respectively. In addition, both simulation and experimental results revealed that the flow resistance at the sample enrichment module is adjustable by punching different outlets, allowing over 50% of the aqueous phase to be removed. YOLOv8n-based droplet detection algorithms realized the counting of cells/beads in droplets, statistically demonstrating single-cell and bead encapsulation rates of 72.2% and 79.2%, respectively. All the results indicate that this on-chip sample enrichment approach can be further developed and employed as a critical component in single-cell encapsulation in water-in-oil droplets.

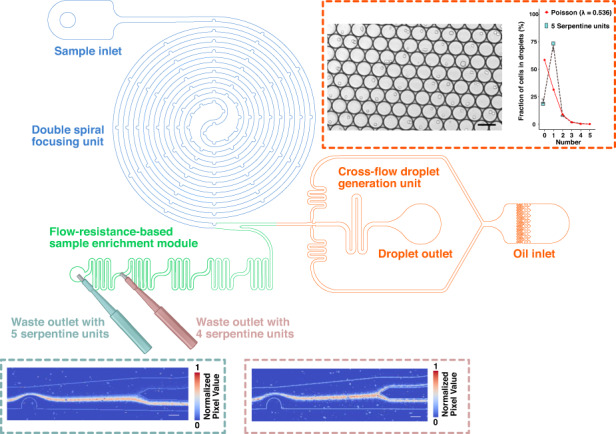

## Introduction

Traditional biomedical studies rely largely on analyzing populations of cells that fail to capture the heterogeneity among a population but simply accept the averaged properties of cell ensembles. Recent efforts have increasingly recognized the importance and significance of studying biological samples at the individual cell level^1–3^. The assumption of the homogeneity of a population of cells has been challenged, and appropriate tools were developed to study the heterogeneity and diversity that exists among individual cells within a tissue. This need to study the spatial heterogeneity of cells in terms of genetic, epigenetic, and morphological traits has driven the development of droplet microfluidic chips for the isolation of individual cells at single-cell resolution^4,5^. Compartmentalizing single cells into each individual water-in-oil emulsion droplet has revolutionized genomic, transcriptomics, and proteomics studies^6,7^. Droplet microfluidics has immense potential in single-cell sequencing^8^, cell lineage tracing^9^, assay development, and drug screening^10^, as well as rare cell analysis^11^.

The single-cell encapsulation rate in traditional droplet microfluidic devices is strongly influenced by the initial cell density in the aqueous phase and the microchannel geometry^12^. In random encapsulation, cells are randomly distributed within the aqueous phase, resulting in a theoretical maximum of 37% droplets encapsulating a single cell, according to the Poisson distribution^12,13^. This low probability leads to a substantial waste of both sample and droplet materials. Therefore, to address this issue, spiral microfluidics are often selected for focusing cells into a single line with equal spacing^12,14–16^, enabling sequential entry of single cells into droplets. For instance, Park et al.^17^ employed a five-loop spiral channel of 140 μm width to focus 15 μm beads into a line and achieved an ~60% encapsulation rate of single beads when *λ* = 0.8 (*λ* is the average number of cells/beads per droplet, *λ* = (Cell/bead suspension density) × (Droplet volume)). While a higher cell density could theoretically increase the encapsulation rate, doubling the suspension density to *λ* = 1.6 did not significantly increase the single-bead encapsulation rate (65%)^17^. This result could be attributed to the decreasing performance of spiral channels on high-concentration samples due to interactions with each other in flow conditions^18,19^. In contrast, Kemna et al.^15^ realized a high yield (77%) of single-cell droplet encapsulation with a similar five-loop spiral channel. Comparing both works, the higher single-cell encapsulation rate should come from the high-concentration cells (*λ* = 1.1) and a narrow channel (50 μm wide); here, narrow spiral channels might contribute to the cell focusing on a high concentration, but resulting in clogging can be a significant drawback. Successful single-cell encapsulation always faces an inherent trade-off between the focusing efficiency and encapsulation rate for a given set of parameters, such as the initial cell density, volumetric flow rate, channel height and width. Droplet formation and encapsulated cell number per droplet are extremely sensitive to these parameters.

**Fig. 1:**
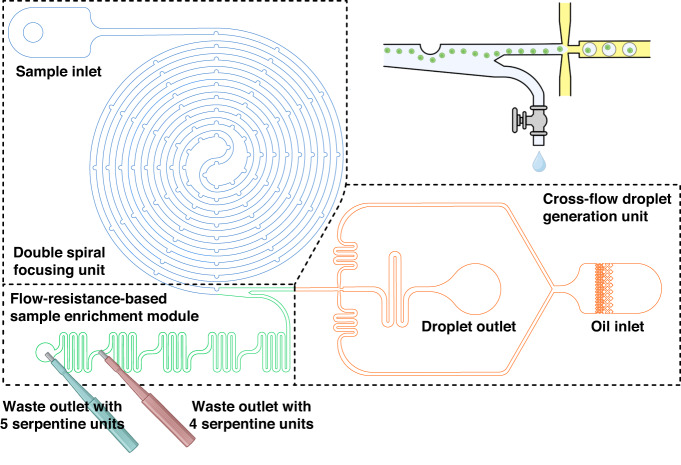
Schematic representation of the microfluidic platform. The cell suspension is initially focused into a single line in the double spiral focusing unit. Excess aqueous phase is then removed from the waste outlets, and the amount of removal is determined by the number of serpentine units used in the sample enrichment module. After sample enrichment, cells are encapsulated into droplets one by one in the droplet generation unit. (Partially made using BioRender.com under premium membership)

Here, we present a novel droplet microfluidic device (Fig. 1) that has a double spiral focusing unit and an on-chip sample enrichment module integrated for consistent and high-throughput single-cell encapsulation. On-chip sample enrichment just before droplet encapsulation avoids needing a high cell density, which risks clogging the narrow channels. We also found that being able to use a lower initial cell concentration reduces the interaction between cells, significantly facilitating cell focusing and single-cell encapsulation. The flow resistance-based sample enrichment module enables adjustment of the cell density after cell focusing by removing the excess aqueous phase. The amount of aqueous phase removed is controllable by altering the number of serpentine units, which is determined by the PDMS chip puncher. Low-density samples and larger channels enhanced the focusing efficiency, reduced the flow shear stress and lowered the possibility of clogging. This novel device significantly increased the flexibility in parameter selection for single-cell encapsulation in water-in-oil droplets.

## Materials and methods

### Device design and fabrication

The microfluidic platform features three units (see Fig. 1): (1) an 8-loop double spiral focusing unit with equally spaced pillars. The spiral channel has a width of 100 μm, and its curvature radius varies between 333 μm for the most inner loop and 3800 μm for the most outer loop. The pillars in this unit are in the shape of a 70 μm half circle and are placed on the inner channel side of the loop every 1/6 loop (30°). The spiral channel was designed based on the previous research of Shen et al.^20,21^ and our group^22–24^. (2) A flow resistance-based sample enrichment module. This module consists of 5 identical serpentine units, each measuring 700 μm in length and 100 μm in width, with a spacing of 700 μm between each serpentine unit. (3) A droplet generation unit, which features a traditional crossflow structure^25–28^. Additional detailed parameters for the microfluidic platform can be found in Fig. S1 in the supplementary materials.

The SU-8 mold with patterns at a depth of 60 μm was fabricated using standard photolithography technology tools in the Mechanobiology Institute’s Nano and Microfabrication Core at the National University of Singapore. The microfluidic chip was then cast from the SU-8 photoresist mold using PDMS (Sylgard 184, Dow Corning, USA) and crosslinked at a 10:1 ratio. For the purpose of evaluating the effect of chip stiffness on the experimental results, a stiffer chip was also fabricated using a 5:1 ratio. Inlets and outlets were punched using a 1.5 mm puncher (Miltex, Integra Life Sciences, USA). A total PDMS chip was realized via PDMS-to-PDMS bonding through the use of oxygen plasma. After bonding, the microchannel was treated with 1H,1H,2H,2H-perfluorododecyltrichlorosilane (370533, Sigma)^29^ to improve the hydrophobicity of the channel wall. The device was then stored in an oven at 60 °C for more than 12 hours to enhance the hydrophobicity. Prior to the experiments, the channel walls were further treated with the superhydrophobic solution (MesoPhobic-2000; MesoBioSystem, China)^30^.

### Sample process

The human metastatic breast cancer cell line MDA-MB-231 and gastric cancer cell line MKN-45 were obtained from American Type Culture Collection (ATCC) and Riken Cell Bank (Tsukuba, Japan), respectively. MDA-MB-231 cells were cultured in standard Dulbecco’s modified Eagle’s medium (DMEM) (Gibco, CA, USA) supplemented with 10% heat-inactivated fetal bovine serum (FBS) (Gibco, CA, USA) and 200 U/mL gentamycin (Gibco, CA, USA). MKN-45 cells were cultured in Roswell Park Memorial Institute (RPMI) 1640 medium containing 10% heat-inactivated fetal bovine serum (FBS) (Gibco, CA, USA) and 1% penicillin‒streptomycin (Gibco, CA, USA). Both cell lines were maintained at 37 °C in a 5% CO_2_ atmosphere and grown to 90% confluence before the experiment.

Prior to droplet encapsulation, MDA-MB-231 or MKN-45 cells were digested with TrypLE^TM^ Express (Gibco, CA, USA) and centrifuged at 200 × g for 3 minutes to remove any remaining digesting agent. The pellet was resuspended in the same culture media to reach the desired concentrations of 1 × 10^6^, 2 × 10^6^, and 4 × 10^6^ cells/mL for experimentation. To evaluate cell viability after droplet encapsulation, droplets were treated with an anti-static gun (Milty Zerostat 3, Armourhome)^31^. This treatment induced droplet merging and allowed the retrieval of a cell suspension. A Trypan blue solution (4%) was used as a cell stain to assess the viability of the retrieved cells, and the results are shown in Fig. S2 in the supplementary materials.

Additionally, polystyrene beads (EPRUI, China) with diameters of 10 μm and 15 μm were utilized as a reference model. To accurately reproduce the conditions pertaining to cell droplet encapsulation, the beads were suspended in identical culture media as employed in the cell experiments, ensuring concentrations of 1 × 10^6^, 2 × 10^6^, and 4 × 10^6^ beads/mL.

### Droplet encapsulation

A bioinert fluorocarbon oil (Novec 7500, 3 M, USA) containing 2% (w/w) surfactant Pico-Surf (Sphere Fluidics, UK) was used as the continuous oil phase. Sample suspensions and oil were injected using separate syringe pumps (Harvard Apparatus, MA, USA). The aqueous phase, namely, cell/bead suspensions, was set at a flow rate of 80 μL/min, while the oil phase flow rate was set at 70 μL/min, unless otherwise specified. Droplets containing beads/cells were produced at a speed of 4000 droplets per second.

### Video processing

All experiments were conducted on an inverted microscope (Olympus IX71, Japan). Cell focusing and excess aqueous phase removal were visualized with a 5× objective lens, while droplet encapsulation was observed using a 10× objective lens. Experimental video clips were recorded using a high-speed camera (FASTCAM SA3, Photron, Japan) set at 2000 frames per second (fps) and a 0.02 ms exposure time.

The standard deviation analysis^17,32^ employed in this study was based on lab-developed Python codes and was used to visualize the trajectory of cells/beads by stacking 1000 high-speed camera frames into one.

Droplet detection and cell/particle counting within the droplets were performed using lab-developed Python code. The Hough gradient method in the OpenCV library^33^ was used to detect droplets and measure their diameters in the microchannel. To count the number of cells/beads in each droplet, a deep learning model (YOLOv8n)^34^ was trained using the droplet datasets generated in this study and employed to analyze all videos. The detected bead/cell numbers were labeled in video clips and can be viewed in Movies S1 and S2, respectively, in the supplementary materials. The source code and trained model are available in the supplementary materials.

## Results

### Bead focusing and enrichment

**Fig. 2:**
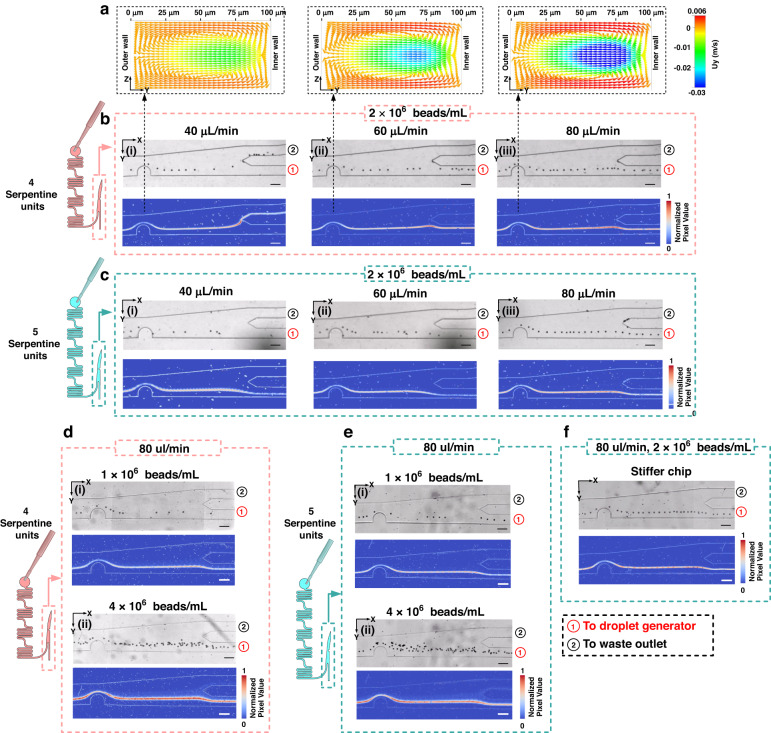
Performance of trajectory focusing and sample enrichment on a chip with 15 μm polystyrene beads at three distinct flow rates: 40, 60, and 80 μL/min and at 3 bead concentrations: 1 × 10^6^, 2 × 10^6^, and 4 × 10^6^ beads/mL. **a** Numerical simulation of the dean flow along the *Y* axis in the narrowest area around the pillar. **b**, **c** Bead trajectories at different flow rates on a chip with **b** 4 serpentine units and **c** 5 serpentine units. **d**, **e** Bead trajectories at varying bead concentrations on a chip with **d** 4 or **e** 5 serpentine units. **f** Bead trajectory in a stiffer PDMS chip. The upper portion of **b**–**f** is the video frames, while the lower portion is the standard deviation plots obtained by overlapping 1000 consecutive frames to illustrate the trajectory and focusing performance. All scale bars indicate 100 μm

Experiments were initially conducted using 15 μm polystyrene beads. Figure 2 illustrates the trajectory of these beads as they moved from the final pillar in the focusing unit to the bifurcation point situated within the sample enrichment module. At the bifurcation point, the subchannel (channel 1) along the inner channel wall leads to the droplet generation unit, while the subchannel (channel 2) on the outer channel wall is directed toward the waste outlet. The resistance pressure in channel 1 remains constant, owing to the steady oil flow rate of 70 μL/min. Conversely, the resistance of the waste outlet (channel 2) depends on the number of serpentines utilized. The resistance difference between the two subchannels determines the amount of removed aqueous phase. In the optimized configuration, all focused beads should flow adjacent to the inner channel wall, entering channel 1 and proceeding to the droplet generation unit. Meanwhile, the excess aqueous phase is eliminated via channel 2, directed to the waste outlet.

Figure 2a displays the Dean flow acceleration along the Y-axis within the narrowest regions of the final pillar at three distinct flow rates: 40, 60, and 80 μL/min. Numerical simulations were carried out using FLOW modules of the ESI-CFD (V2016.0, ESI-CFD, Inc., Huntsville, AL, USA), with comprehensive simulation steps detailed in our previous work^21,35^. The simulation results showed that an increase in flow rate amplifies the Dean flow, subsequently enhancing the Dean drag force and promoting the lateral migration of objects (cells/beads) toward the inner channel wall. These simulations also provided insights into the force distribution and potential positions of the forced beads/cells that would be focused.

The impact of flow rate on the object focusing is shown in Fig. 2b, c. At a flow rate of 40 μL/min, the focused bead line tended to be dragged into channel 2 (waste) when using 4 serpentine units (see Fig. 2b(i)). To redirect the beads into channel 1, increasing the flow rate proved effective, as it provided a greater Dean drag force that focused the beads closer to the inner wall, as demonstrated in Fig. 2b(ii, iii). Alternatively, increasing the flow resistance at the waste outlet by employing 5 serpentine units achieved a similar result at the expense of reduced aqueous phase removal (see Fig. 2c). Beyond the focusing performance, a high flow rate (e.g., 80 μL/min) also mitigated the object sedimentation in the platform. For instance, the continuous beads flow in Fig. 2c(iii) was better than that in Fig. 2c(I, ii), despite the constant original concentration. Consequently, a flow rate of 80 μL/min was utilized for subsequent experiments because it successfully guided beads into channel 1 and realized a more continuous beads flow.

We maintained the flow rate at 80 μL/min and varied the bead concentration (1 × 10^6^ and 4 × 10^6^ beads/mL), as shown in Figs. 2d, e. Compared to the concentration of 2 × 10^6^ beads/mL (Fig. 2b(iii), c(iii)), a lower concentration (1 × 10^6^ beads/mL) of beads was also successfully focused into a line, but the distance between each bead was less stable. A higher concentration (4 × 10^6^ beads/mL) presented a more severe problem; the bead-to-bead interaction nearly caused the failure of focusing within the spiral channel, and the beads could not be focused into a single file. Therefore, the optimized concentration was set at 2 × 10^6^ particles/mL.

Additionally, we studied the impact of the stiffness of the microfluidic chip, with the results shown in Fig. 2f. By mixing the elastomer base and curing agent at a ratio of 5:1, a stiffer PDMS chip was obtained compared to the commonly used 10:1 ratio. In terms of bead focusing and sample enrichment, no difference was observed between Fig. 2c(iii), f, indicating that the stiffness of the chip did not significantly impact these parameters.

**Fig. 3:**
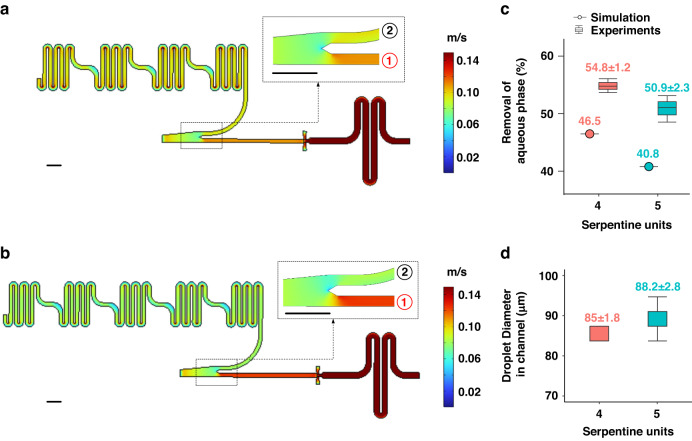
Sample enrichment module analysis. **a**, **b** Simulation results of the flow rate distribution on a chip consisting of **a** 4 and **b** 5 serpentine units. The scale bar indicates 400 μm. **c** Aqueous removal efficiency in simulation and experiments. **d** Droplet diameter in the channel in the experiments. The mean values along with the standard deviations for the experimental data are labeled above each corresponding boxplot

The sample enrichment efficiency was evaluated through simulation and experiments, as depicted in Fig. 3. The simulation^36^ was conducted in a two-dimensional domain using water as the aqueous phase and Fluo-Oil 7500 as the oil phase, utilizing COMSOL Multiphysics 5.3a. All parameters were consistent with those in the experiments; specifically, the flow rates for the aqueous and oil phases were 0.111 m/s and 0.097 m/s, respectively. By increasing the serpentine units from 4 to 5, there was an apparent increase in the flow rate in the channel connected to the droplet generation unit, as seen in Fig. 3a, b. Statistically, in Fig. 3c, more serpentine units led to a smaller proportion of removed aqueous phase, decreasing from 46.5% to 40.8%.

In the experiments, the removal efficiency was quantified by weighing the aqueous phase expelled from the waste outlet within 1 minute, with each measurement repeated three times. The distribution of the experimental data is displayed in boxplots, with detailed mean values and standard deviations labeled above each corresponding boxplot. Additionally, all data represented in the boxplots can be found in the corresponding tables in the supplementary materials. Similar experimental results to the simulation were obtained: 54.8% of the aqueous phase was removed when using 4 serpentine units, compared to 50.9% with five serpentine units in Fig. 3c. This configuration led to an increase in the droplet diameter on the chip (Fig. 3d) from ~85 μm to 88.2 μm. In these results, a difference in the specific amount of removed phase was observed, as more aqueous phase was removed in the experiments than in the simulation. This might occur because the simulation could not perfectly replicate all the parameters and conditions in the experiments. However, both results support the conclusion that the removal of the aqueous phase can be controlled by changing the number of serpentine units utilized.

### Droplet encapsulation of beads

**Fig. 4:**
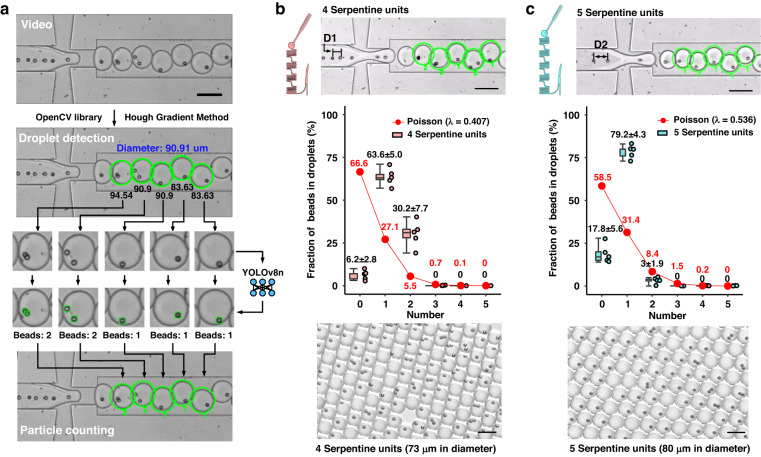
YOLOv8n-based object detection in droplets and the detected results of bead encapsulation at the optimized experimental parameters: aqueous phase: 80 μL/min, oil phase: 70 μL/min, bead concentration: 2 × 10^6^ beads/mL. **a** A flowchart illustrates the video processing algorithm for detecting droplets and counting the beads within each droplet. **b**, **c** These panels show the distribution of bead numbers within droplets for platforms with **b** 4 and **c** 5 serpentine units. All scale bars are set at 100 μm. The mean values along with the standard deviations for the experimental data are labeled above each corresponding boxplot

After sample enrichment on the chip, the bead flow was directed to the droplet generation unit, where beads sequentially entered the droplets, as depicted in Fig. 4. To facilitate data analysis, a lab-developed video processing program was employed (see Fig. 4a). Specifically, the Hough gradient method was utilized to detect droplets and measure their diameters in each video frame, labeling them with green circles. Subsequently, the detected droplets were analyzed by a trained YOLOv8n model (available in the supplementary materials), allowing for the detection and counting of all cells or beads within each droplet. This counted number is also displayed below each droplet in the frame.

Realization of sample enrichment on-chip is evidenced by Fig. 4b, c. Four serpentine units removed more aqueous phase, leading to a higher bead concentration at the droplet generation unit, thereby reducing the distance between beads and increasing the likelihood of encapsulation by more than one bead per droplet. Due to the channel depth of 60 μm, all droplets within the chip experienced compression. The actual diameter of 100 droplets was measured outside the chip, yielding ~73 μm (±0.2689) and 80 μm (±0.3455) for four and five serpentine units, respectively. These measurements correspond to *λ* values of 0.407 and 0.536, respectively. The Poisson distribution was calculated via Eq. (1), where *k* is the number of objects in the droplet and *λ* is the average number of cells per droplet.

1 $$P\left(\lambda ,k\right)=\frac{{\lambda }^{k}{e}^{-\lambda }}{k!}$$

The boxplot (see Fig. 4b, c) represents the fraction of beads in droplets obtained by counting beads in a continuous sequence of 100 droplets in a video and repeating the quantification five times for the following droplets. In Fig. 4b, four serpentine units led to fewer empty droplets (6.2%) compared to 66.6% in the Poisson distribution. However, the high concentration after the on-chip enrichment also resulted in 30.2% of droplets encapsulating two beads, which is higher than the 5.5% in the Poisson distribution. In contrast, Fig. 4c shows a high-efficiency single-bead encapsulation rate of 79.2% by removing less aqueous phases via 5 serpentine units. Meanwhile, the double-bead case was reduced to 3%. This phenomenon is intriguing, as the same initial concentration can yield different single-/multiple-bead encapsulation results by altering the outlet resistance simply through punching outlets at various locations. This effect highlights the flexibility and potential for optimization in microfluidic systems for various applications.

**Fig. 5:**
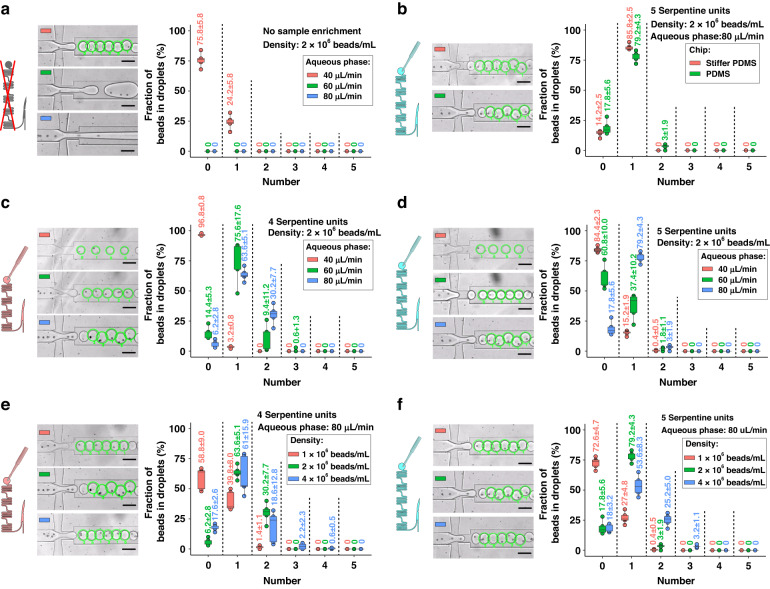
Droplet encapsulation of 15 μm polystyrene beads. **a** Droplet generation on a chip without the sample enrichment module at flow rates of 40, 60, and 80 μL/min. **b** Impact of chip stiffness on generation and encapsulation rate. **c**, **d** Effect of aqueous phase flow rate (i.e., 40, 60, 80 μL/min) on a chip with **c** 4 and **d** 5 serpentine units. **e**, **f** Influence of bead concentration (i.e., 1 × 10^6^, 2 × 10^6^, and 4 × 10^6^ beads/mL) on a chip with **e** 4 and **f** 5 serpentine units. All scale bars are set at 100 μm. The mean values along with the standard deviations for the experimental data are labeled above each corresponding boxplot

Figure 5 illustrates the variations in droplet generation and encapsulation rate based on key experimental parameters such as flow rate (40, 60, 80 μL/min), bead concentration (1 × 10^6^, 2 × 10^6^, and 4 × 10^6^ beads/mL), and chip stiffness. First, as depicted in Fig. 5a, the spiral channel is directly connected to the droplet generation unit without any removal of the aqueous phase, restricting droplet generation to a low rate of 40 μL/min (aqueous phase). This condition leads to significant sample sedimentation and a discontinuous line of focused beads. Additionally, in the absence of sample enrichment, ~75.8% of droplets remained empty at a bead concentration of 2 × 10^6^ beads/mL. For the stiffness of the PDMS chip, in Fig. 5b, there appears to be no significant difference caused by the chip stiffness.

Figure 5c, d summarizes the encapsulation efficiency at different flow rates using 4 and 5 serpentine units, respectively. The combination of a sample flow rate of 60 µl/min, a density of 2 × 10^6^ beads/mL, and 4 serpentine units appears to be the optimal set of parameters for single-bead encapsulation (75.6% in Fig. 5c). However, the distance between beads at this flow rate is unstable (see Fig. 2b(ii)), resulting in significant variation in the encapsulation rate and, consequently, a larger error bar compared to the encapsulation rate at a higher flow rate (80 µl/min). For more stable droplet encapsulation, a sample flow rate of 80 µl/min was determined as the optimal choice, enabling a stable encapsulation of up to 79.2% (Fig. 5d).

Figure 5e, f depicts the encapsulation efficiency at varying bead densities using 4 and 5 serpentine units, respectively. The concentration of 2 × 10^6^ beads/mL is notably more efficient in single-bead droplet encapsulation than either 1 × 10^6^ beads/mL or 4 × 10^6^ beads/mL. Utilizing a lower concentration of 1 × 10^6^ beads/mL led to the generation of >50% empty droplets in both experimental setups. This inefficiency at a lower concentration can be attributed to the inability to focus the beads into a single-file arrangement with uniform spacing, corroborating the data presented in Fig. 2d, e. Conversely, at a high concentration of 4 × 10^6^ beads/mL, ~30% of the droplets contained multiple beads, accompanied by a notably larger error bar, as shown in Fig. 5e, f. This phenomenon is attributed to the excessive bead density overwhelming the capacity of the spiral channel to focus beads into a single-file arrangement (as illustrated in Fig. 2d, e). Consequently, multiple beads arrived at the droplet generation unit simultaneously, leading to the encapsulation of more than one bead per droplet.

### Cell focusing and enrichment

**Fig. 6:**
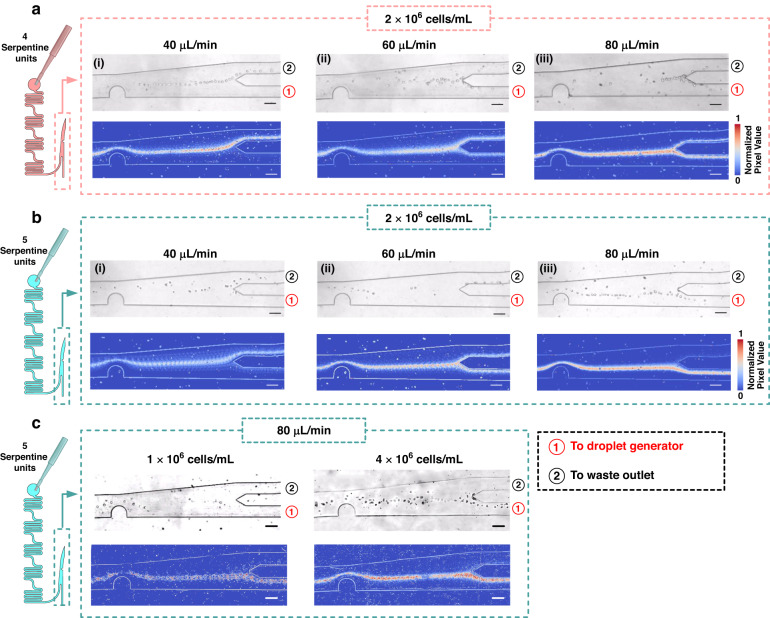
Performance of trajectory focusing and sample enrichment on chip with cells (MDA-MB-231) at three distinct flow rates: 40, 60, and 80 μL/min and at three cell concentrations: 1 × 10^6^, 2 × 10^6^, and 4 × 10^6^ beads/mL. **a**, **b** Cell trajectories at different flow rates on a chip with **a** 4 serpentine units and **b** 5 serpentine units. **c** Cell trajectories at different cell concentrations on a chip with 5 serpentine units. For each subplot, the upper portion consists of video frames, while the lower portion displays standard deviation plots obtained by overlapping 1000 consecutive frames to illustrate the trajectory and focusing performance. All scale bars indicate 100 μm

Figure 6 demonstrates the focusing trajectory and on-chip sample enrichment for cells. In contrast to the 15 μm polystyrene beads, the focusing performance of cells is suboptimal at 40 μL/min (see Fig. 6a(i), b(i)). This result is attributed to the heterogeneity of cells, which increases the difficulty of focusing them into a line compared to the case of polystyrene beads with consistent properties, such as density and morphology. By increasing the flow rate and, subsequently the Dean drag force, cells exhibit improved focusing trajectories (see Fig. 6a(iii) and 6b(iii)). Additionally, Fig. 6a shows that although the proportion of cells entering channel 1 increased with the flow rate, a portion of cells was still lost (see Fig. 6a(iii)). To minimize sample loss, 5 serpentine units are necessary to increase the outlet flow resistance, even though sample loss still occurred at flow rates below 60 μL/min (see Fig. 6b(I, ii)). Ultimately, employing a flow rate of 80 μL/min and 5 serpentine units successfully directs almost all cells into channel 1. When varying the cell concentration, Fig. 6c demonstrates similar results to the bead experiments. A low density of 1 × 10^6^ cells/mL caused the focused cells to be discontinuous. Conversely, a high density of 4 × 10^6^ cells/mL resulted in too many cells to be arranged in a line, leading to a failure in focusing.

### Droplet encapsulation of cells

**Fig. 7:**
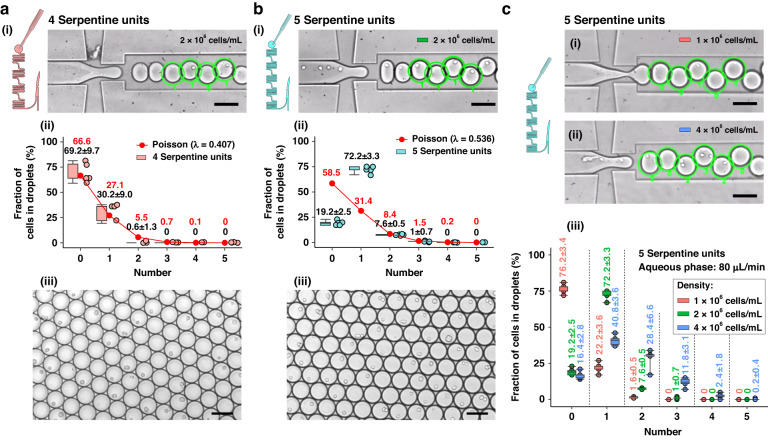
Droplet encapsulation of cells (MDA-MB-231). Distribution of bead number within droplets for platforms with **a** 4 and **b** 5 serpentine units. **c** Influence of cell concentration (i.e., 1 × 10^6^, 2 × 10^6^, and 4 × 10^6^ cells/mL) on the encapsulation efficiency on a chip with 5 serpentine units. All scale bars indicate 100 μm. The mean values along with the standard deviations for the experimental data are labeled above each corresponding boxplot

Figure 7a(i) represents the droplet encapsulation of cells when using a flow rate of 80 μL/min and 4 serpentine units. The boxplot in this case resembles a Poisson distribution coincidentally. Sample loss (see Fig. 6a(iii)) resulted in 69.2% empty droplets (Fig. 7a(ii, iii)), with only 30.2% of droplets containing one cell. Upon the removal of 54.76% of the water from the cell suspension using 4 serpentine units (see Fig. 3c), the focused cells collide at the junction, resulting in a loss of cells. Typically, sample loss is not acceptable in experiments, so an adjustment to 5 serpentine units was applied. This modification increased the flow resistance at the waste outlet and allowed more water (3.72% in Fig. 3c) to be guided into the droplet generation unit. Consequently, almost all cells could be directed into the droplet generation unit, fulfilling the intended design. As a result, 72.2% of droplets contained one cell, slightly lower than the 80% achieved with 15 μm beads (see Fig. 7b(ii, iii)). Another difference when using cells compared to using beads is that the likelihood of generating double-cell droplets was slightly higher (7.6% vs. 3%). This difference might occur because some cells touch the channel wall during sample enrichment, disturbing the equivalent distance between cells. This hypothesis is supported by comparing Figs. 4c and 7b, which show that cells were focused into a line but were quite close to the channel wall, while beads were located at the channel center in the droplet generation unit. The viability of the cells is 92.4% after droplet encapsulation, which indicates that the chip surface treatment does not negatively affect cell viability.

Additionally, similar to the experiments on beads, Fig. 7c illustrates how the cell density in suspension is an important parameter determining the encapsulation rate. A discontinuous line of focused cells at a low density (1 × 10^6^ cells/mL) results in 76.2% empty droplets, while an unfocused line of high-density cells (such as 4 × 10^6^ cells/mL) leads to ~40% of droplets containing more than one cell.

## Discussion

Spiral channel techniques have been widely adopted in droplet microfluidics to organize randomly distributed objects into a well-arranged line before droplet encapsulation. To date, various types of spiral channels^15,34,37,38^ have been designed to enhance both the focusing efficiency and encapsulation rate. Here, we report that the performance of these techniques can be further optimized by incorporating a specific structure, namely, a sample enrichment module, subsequent to the spiral channel. This design ensures that all particles in low-density suspensions are arranged into a line and spaced nearly uniformly, while excess water can be removed after focusing processing but before encapsulation. This process enriches the sample density and reduces the number of empty droplets. Compared to standard droplet techniques, where encapsulation efficiency is primarily determined by suspension density, we provide an alternative approach by controlling the suspension density through a simple microfluidic structure.

The necessity of performing sample enrichment after focusing is demonstrated in Figs. 2 and 6. Regardless of whether cells or beads are used, they can all be focused into a line at a density no greater than 2 × 10^6^ objects/mL. When the suspension density reaches 4 × 10^6^ objects/mL, particle interactions make it difficult for them to align in a line, resulting in a high rate of multiple particle encapsulations. Conversely, when the suspension density is too low (1 × 10^6^ objects/mL), achieving a uniform spacing between particles becomes challenging. In our work, the optimal density is 2 × 10^6^ objects/mL, which can be enriched to nearly 4 × 10^6^ objects/mL after focusing by removal of ~50% of the water (Fig. 3) from the sample flow.

With the integration of the sample enrichment module, we realized high single-particle encapsulation rates: 79.2% for 15 μm beads and 72.2% for MDA-MB-231 cells (~14 μm). This high level of performance is retained across beads or cells with different diameters. As illustrated in Fig. S3, the single-cell encapsulation rate reached 70.4% for MKN-45 cells, which have an approximate diameter of 11 μm. For reference, commercially available polystyrene beads with a similar size to MKN-45 cells are typically 10 μm in diameter. In this context, the single-bead encapsulation rate for 10 μm beads was an impressive 85.2%.

Without the sample enrichment module, the rate dropped to 24.2% for beads (see Fig. 5a). The large difference might be partly attributed to the flow rate. When 50% of the water is removed, the sample suspension can be pumped at a higher rate (i.e., 80 μL/min), while without the sample enrichment module, the flow rate must be limited to 40 μL/min. A higher flow rate is known to exert a greater push force on particles and reduce sample sedimentation during transportation. This hypothesis is supported by Figs. 2b, c and 6a, b, where more cells/beads are observed in the frame at 80 μL/min compared to 40 μL/min.

In our design, sample focusing is an essential step to prevent sample loss when removing excess water. We utilized the spiral channel to carry out this focusing, directing all samples into the droplet generation unit in a sequential manner. Although active focusing techniques such as acoustics^39^ (which also enable highly efficient on-chip cell focusing) might also be applicable to our system, we emphasize that our present design is a simple and integrated on-chip enrichment tool that eliminates the need for additional enrichment steps prior to experimentation.

In subsequent research, this chip can be further refined. Currently, we are operating at one extreme, namely either 4/5 or 5/5 resistors. Future work may redesign the sample enrichment module or simply add more resistors to extend the dynamic range of resistance. Additionally, the chip design could be augmented by combining two or more spiral channels^17,40^. This would enable the coencapsulation of cell-bead^14,41,42^ or cell-cell^43^ pairs, broadening the applicability of the design to serve various purposes.

## Conclusion

In conclusion, this study successfully demonstrates the advantages of integrating an on-chip sample enrichment module into a droplet microfluidic platform, resulting in a more versatile and flexible device for various applications. The innovative approach of punching outlets to control the flow resistance at waste outlets allows for concentration enrichment of focused samples before droplet generation. This method enables the focusing of cells or beads at low concentrations while maintaining high-efficiency single-cell or single-bead encapsulation rates. Future research may focus on refining the design of droplet microfluidic devices to further enhance the focusing performance and sample enrichment efficiency. Such progress would enable more precise control over single or multiple bead or cell encapsulation rates and further expand the range of applications for this versatile platform.

### Supplementary information

Supplementary Masterial

Supplementary video 1

Supplementary video 2

Source code
