# Supplementary material for: Enhancing single-cell encapsulation in droplet microfluidics with fine-tunable on-chip sample enrichment
Source: Microsyst Nanoeng. 2024 Jan 2;10:3. doi: 10.1038/s41378-023-00631-y (PMC10758392; doi:10.1038/s41378-023-00631-y)
Supplement: Supplementary file 1 — Supplementary Masterial [file 41378_2023_631_MOESM1_ESM.docx]

**Enhancing Single-Cell Encapsulation Performance in Droplet Microfluidics with Fine-tunable On-Chip Sample Enrichment**

Tao Tang^‡a^, Hao Zhao^‡a,b^, Shaofei Shen^‡c^, Like Yang^a^, Chwee Teck Lim*^a,d,e,f^

1. Department of Biomedical Engineering, National University of Singapore, 117583, Singapore
2. Integrative Sciences and Engineering Programme, NUS Graduate School, National University of Singapore, 119077, Singapore
3. Shanxi Key Lab for Modernization of TCVM, College of Life Science, Shanxi Agricultural University, Taigu, Shanxi, 030801, China
4. Institute for Health Innovation & Technology, National University of Singapore, 117599, Singapore
5. Mechanobiology Institute, National University of Singapore, 117411, Singapore
6. Institute for Digital Molecular Analytics and Science, Nanyang Technological University, Singapore 636921

*Corresponding author, email: [ctlim@nus.edu.sg](mailto:ctlim@nus.edu.sg)

‡ These authors contributed equally to this work.


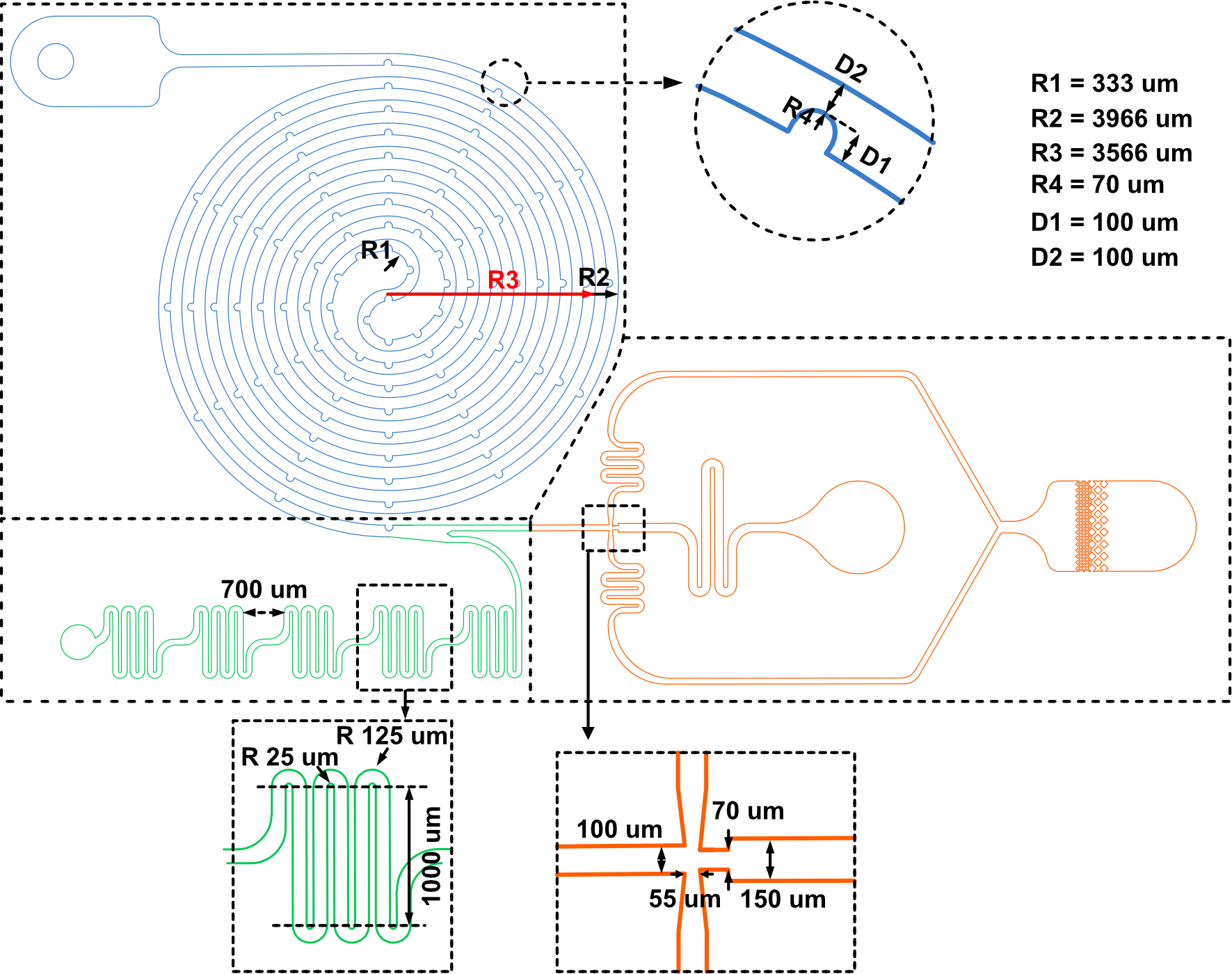


Figure S1: The design of the droplet microfluidic platform with on-chip enrichment


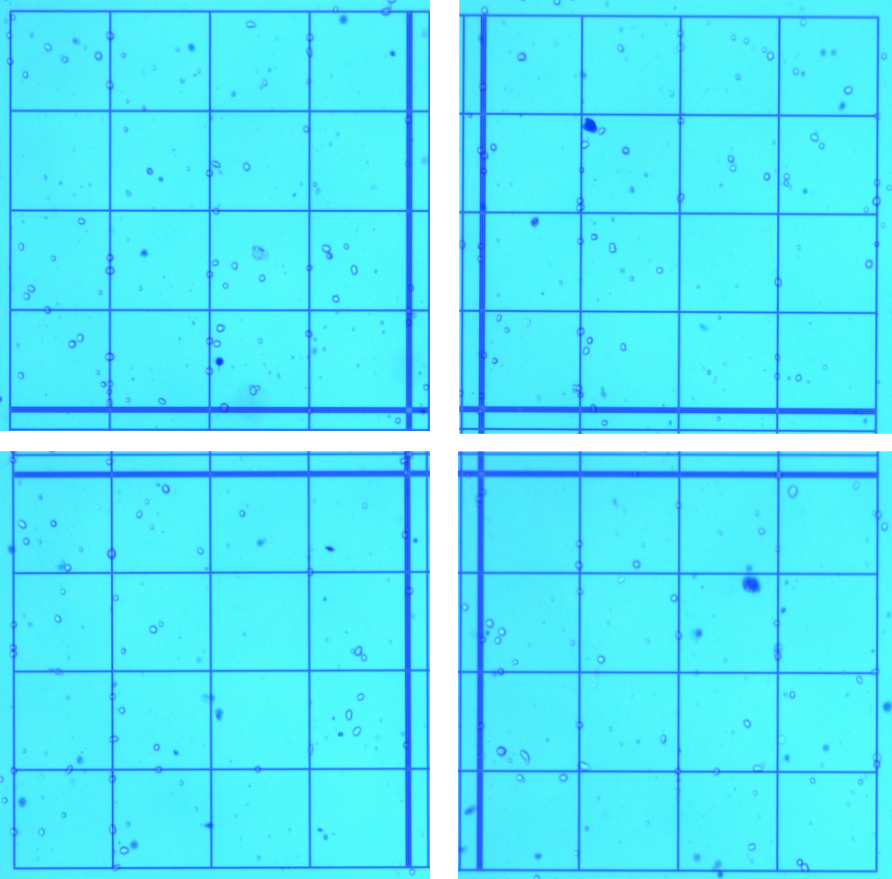


Figure S2: Cell viability after the droplet encapsulation.

MDA-MB-231 cells that were encapsulated in droplets are retrieved immediately by merging the droplets and collecting aqueous phase containing cells. The sample was stained with 0.4% Trypan blue solution and counted with hemocytometer (C-CHIP Neubauer Improved|DHC-N01, NanoEntek). There are 21 dead cells (stained blue) out of 276 cells in total, making a viability of 92.4%


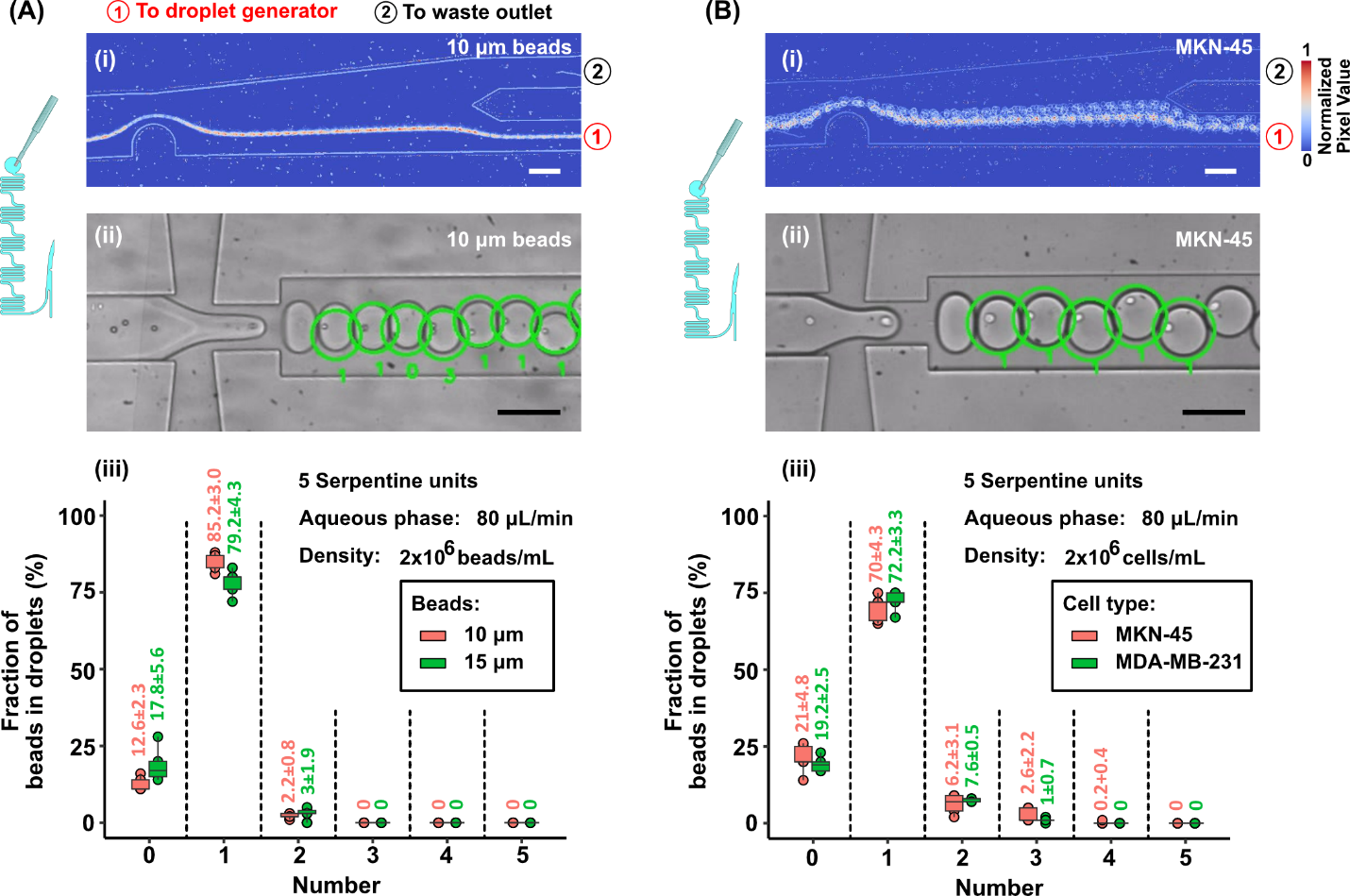


Figure S3: Droplet encapsulation of (A) beads (10 μm) and (B) cells (MKN-45) on the micro-chip with 5 serpentines units. (i) Performance of trajectory focusing and sample enrichment on chip. (ii) Single beads or cells encapsulation in droplets. (iii) Influence of bead size or cell type on the encapsulation rate. All scale bars indicate 100 μm. The mean values along with the standard deviations for the experimental data are labelled above each corresponding boxplot.

Table S1: Parameters used in Figure 3C

| Serpentine Units | Lower  Whisker | Lower Quartile | Median | Upper  Quartile | Upper  Whisker | Mean | SD |
| --- | --- | --- | --- | --- | --- | --- | --- |
| 4 | 53.669222 | 54.216867 | 54.764513 | 55.421687 | 56.078861 | 54.837532 | 1.206478 |
| 5 | 48.521358 | 49.780942 | 51.040526 | 52.081051 | 53.121577 | 50.894487 | 2.303584 |

Table S2: Parameters used in Figure 3D

| Serpentine Units | Lower  Whisker | Lower Quartile | Median | Upper  Quartile | Upper  Whisker | Mean | SD |
| --- | --- | --- | --- | --- | --- | --- | --- |
| 4 | 83.6326 | 83.6326 | 83.6326 | 87.2688 | 87.2688 | 84.9593062 | 1.75070834 |
| 5 | 83.6326 | 87.2688 | 87.2688 | 90.905 | 94.5412 | 88.1788435 | 2.87451121 |

Table S3: Parameters used in Figure 4B

| Bead  Number | Lower  Whisker | Lower Quartile | Median | Upper  Quartile | Upper  Whisker | Mean | SD |
| --- | --- | --- | --- | --- | --- | --- | --- |
| 0 | 3 | 4 | 7 | 7 | 10 | 6.2 | 2.77488739 |
| 1 | 57 | 62 | 63 | 65 | 71 | 63.6 | 5.07937004 |
| 2 | 19 | 28 | 31 | 33 | 40 | 30.2 | 7.66159252 |
| 3 | 0 | 0 | 0 | 0 | 0 | 0 | 0 |
| 4 | 0 | 0 | 0 | 0 | 0 | 0 | 0 |
| 5 | 0 | 0 | 0 | 0 | 0 | 0 | 0 |

Table S4: Parameters used in Figure 4C

| Bead  Number | Lower  Whisker | Lower Quartile | Median | Upper  Quartile | Upper  Whisker | Mean | SD |
| --- | --- | --- | --- | --- | --- | --- | --- |
| 0 | 14 | 15 | 17 | 20 | 28 | 17.8 | 5.6302753 |
| 1 | 72 | 76 | 80 | 80 | 83 | 79.2 | 4.2661458 |
| 2 | 0 | 3 | 3 | 4 | 5 | 3 | 1.87082869 |
| 3 | 0 | 0 | 0 | 0 | 0 | 0 | 0 |
| 4 | 0 | 0 | 0 | 0 | 0 | 0 | 0 |
| 5 | 0 | 0 | 0 | 0 | 0 | 0 | 0 |

Table S5: Parameters used in Figure 5A

| Bead  Number | Flow Rate  (μL/min) | Lower  Whisker | Lower Quartile | Median | Upper  Quartile | Upper  Whisker | Mean | SD |
| --- | --- | --- | --- | --- | --- | --- | --- | --- |
| 0 | 40 | 83 | 88 | 88 | 90 | 94 | 88.6 | 3.97492138 |
| 0 | 60 | 0 | 0 | 0 | 0 | 0 | 0 | 0 |
| 0 | 80 | 0 | 0 | 0 | 0 | 0 | 0 | 0 |
| 1 | 40 | 6 | 9 | 12 | 12 | 16 | 11 | 3.74165739 |
| 1 | 60 | 0 | 0 | 0 | 0 | 0 | 0 | 0 |
| 1 | 80 | 0 | 0 | 0 | 0 | 0 | 0 | 0 |
| 2 | 40 | 0 | 0 | 0 | 1 | 1 | 0.4 | 0.54772256 |
| 2 | 60 | 0 | 0 | 0 | 0 | 0 | 0 | 0 |
| 2 | 80 | 0 | 0 | 0 | 0 | 0 | 0 | 0 |
| 3 | 40 | 0 | 0 | 0 | 0 | 0 | 0 | 0 |
| 3 | 60 | 0 | 0 | 0 | 0 | 0 | 0 | 0 |
| 3 | 80 | 0 | 0 | 0 | 0 | 0 | 0 | 0 |
| 4 | 40 | 0 | 0 | 0 | 0 | 0 | 0 | 0 |
| 4 | 60 | 0 | 0 | 0 | 0 | 0 | 0 | 0 |
| 4 | 80 | 0 | 0 | 0 | 0 | 0 | 0 | 0 |
| 5 | 40 | 0 | 0 | 0 | 0 | 0 | 0 | 0 |
| 5 | 60 | 0 | 0 | 0 | 0 | 0 | 0 | 0 |
| 5 | 80 | 0 | 0 | 0 | 0 | 0 | 0 | 0 |

Table S6: Parameters used in Figure 5B

| Bead  Number | Chip | Lower  Whisker | Lower Quartile | Median | Upper  Quartile | Upper  Whisker | Mean | SD |
| --- | --- | --- | --- | --- | --- | --- | --- | --- |
| 0 | Stiffer | 10 | 14 | 15 | 16 | 16 | 14.2 | 2.48997992 |
| 0 | Normal | 14 | 15 | 17 | 20 | 28 | 17.8 | 5.6302753 |
| 1 | Stiffer | 84 | 84 | 85 | 86 | 90 | 85.8 | 2.48997992 |
| 1 | Normal | 72 | 76 | 80 | 80 | 83 | 79.2 | 4.2661458 |
| 2 | Stiffer | 0 | 0 | 0 | 0 | 0 | 0 | 0 |
| 2 | Normal | 0 | 3 | 3 | 4 | 5 | 3 | 1.87082869 |
| 3 | Stiffer | 0 | 0 | 0 | 0 | 0 | 0 | 0 |
| 3 | Normal | 0 | 0 | 0 | 0 | 0 | 0 | 0 |
| 4 | Stiffer | 0 | 0 | 0 | 0 | 0 | 0 | 0 |
| 4 | Normal | 0 | 0 | 0 | 0 | 0 | 0 | 0 |
| 5 | Stiffer | 0 | 0 | 0 | 0 | 0 | 0 | 0 |
| 5 | Normal | 0 | 0 | 0 | 0 | 0 | 0 | 0 |

Table S7: Parameters used in Figure 5C

| Bead  Number | Flow Rate  (μL/min) | Lower  Whisker | Lower Quartile | Median | Upper  Quartile | Upper  Whisker | Mean | SD |
| --- | --- | --- | --- | --- | --- | --- | --- | --- |
| 0 | 40 | 96 | 96 | 97 | 97 | 98 | 96.8 | 0.83666003 |
| 0 | 60 | 11 | 11 | 11 | 16 | 23 | 14.4 | 5.27257053 |
| 0 | 80 | 3 | 4 | 7 | 7 | 10 | 6.2 | 2.77488739 |
| 1 | 40 | 2 | 3 | 3 | 4 | 4 | 3.2 | 0.83666003 |
| 1 | 60 | 48 | 68 | 87 | 87 | 88 | 75.6 | 17.5584737 |
| 1 | 80 | 57 | 62 | 63 | 65 | 71 | 63.6 | 5.07937004 |
| 2 | 40 | 0 | 0 | 0 | 0 | 0 | 0 | 0 |
| 2 | 60 | 1 | 2 | 2 | 16 | 26 | 9.4 | 11.171392 |
| 2 | 80 | 19 | 28 | 31 | 33 | 40 | 30.2 | 7.66159252 |
| 3 | 40 | 0 | 0 | 0 | 0 | 0 | 0 | 0 |
| 3 | 60 | 0 | 0 | 0 | 0 | 3 | 0.6 | 1.34164079 |
| 3 | 80 | 0 | 0 | 0 | 0 | 0 | 0 | 0 |
| 4 | 40 | 0 | 0 | 0 | 0 | 0 | 0 | 0 |
| 4 | 60 | 0 | 0 | 0 | 0 | 0 | 0 | 0 |
| 4 | 80 | 0 | 0 | 0 | 0 | 0 | 0 | 0 |
| 5 | 40 | 0 | 0 | 0 | 0 | 0 | 0 | 0 |
| 5 | 60 | 0 | 0 | 0 | 0 | 0 | 0 | 0 |
| 5 | 80 | 0 | 0 | 0 | 0 | 0 | 0 | 0 |

Table S8: Parameters used in Figure 5D

| Bead  Number | Flow Rate  (μL/min) | Lower  Whisker | Lower Quartile | Median | Upper  Quartile | Upper  Whisker | Mean | SD |
| --- | --- | --- | --- | --- | --- | --- | --- | --- |
| 0 | 40 | 82 | 83 | 84 | 85 | 88 | 84.4 | 2.30217289 |
| 0 | 60 | 52 | 55 | 55 | 66 | 76 | 60.8 | 10.034939 |
| 0 | 80 | 14 | 15 | 17 | 20 | 28 | 17.8 | 5.6302753 |
| 1 | 40 | 12 | 15 | 16 | 16 | 17 | 15.2 | 1.92353841 |
| 1 | 60 | 22 | 32 | 42 | 45 | 46 | 37.4 | 10.2371871 |
| 1 | 80 | 72 | 76 | 80 | 80 | 83 | 79.2 | 4.2661458 |
| 2 | 40 | 0 | 0 | 0 | 1 | 1 | 0.4 | 0.54772256 |
| 2 | 60 | 0 | 2 | 2 | 2 | 3 | 1.8 | 1.09544512 |
| 2 | 80 | 0 | 3 | 3 | 4 | 5 | 3 | 1.87082869 |
| 3 | 40 | 0 | 0 | 0 | 0 | 0 | 0 | 0 |
| 3 | 60 | 0 | 0 | 0 | 0 | 0 | 0 | 0 |
| 3 | 80 | 0 | 0 | 0 | 0 | 0 | 0 | 0 |
| 4 | 40 | 0 | 0 | 0 | 0 | 0 | 0 | 0 |
| 4 | 60 | 0 | 0 | 0 | 0 | 0 | 0 | 0 |
| 4 | 80 | 0 | 0 | 0 | 0 | 0 | 0 | 0 |
| 5 | 40 | 0 | 0 | 0 | 0 | 0 | 0 | 0 |
| 5 | 60 | 0 | 0 | 0 | 0 | 0 | 0 | 0 |
| 5 | 80 | 0 | 0 | 0 | 0 | 0 | 0 | 0 |

Table S9: Parameters used in Figure 5E

| Bead  Number | Density  (beads/mL) | | Lower  Whisker | Lower Quartile | Median | Upper  Quartile | Upper  Whisker | Mean | SD |
| --- | --- | --- | --- | --- | --- | --- | --- | --- | --- |
| 0 | | 1.00E+06 | 48 | 50 | 64 | 65 | 67 | 58.8 | 9.03880523 |
| 0 | | 2.00E+06 | 3 | 4 | 7 | 7 | 10 | 6.2 | 2.77488739 |
| 0 | | 4.00E+06 | 14 | 17 | 17 | 19 | 21 | 17.6 | 2.60768096 |
| 1 | | 1.00E+06 | 33 | 34 | 35 | 47 | 50 | 39.8 | 8.04363102 |
| 1 | | 2.00E+06 | 57 | 62 | 63 | 65 | 71 | 63.6 | 5.07937004 |
| 1 | | 4.00E+06 | 44 | 52 | 53 | 77 | 79 | 61 | 15.9216833 |
| 2 | | 1.00E+06 | 0 | 1 | 1 | 2 | 3 | 1.4 | 1.14017543 |
| 2 | | 2.00E+06 | 19 | 28 | 31 | 33 | 40 | 30.2 | 7.66159252 |
| 2 | | 4.00E+06 | 4 | 6 | 24 | 27 | 32 | 18.6 | 12.7593103 |
| 3 | | 1.00E+06 | 0 | 0 | 0 | 0 | 0 | 0 | 0 |
| 3 | | 2.00E+06 | 0 | 0 | 0 | 0 | 0 | 0 | 0 |
| 3 | | 4.00E+06 | 0 | 0 | 2 | 4 | 5 | 2.2 | 2.28035085 |
| 4 | | 1.00E+06 | 0 | 0 | 0 | 0 | 0 | 0 | 0 |
| 4 | | 2.00E+06 | 0 | 0 | 0 | 0 | 0 | 0 | 0 |
| 4 | | 4.00E+06 | 0 | 0 | 1 | 1 | 1 | 0.6 | 0.54772256 |
| 5 | | 1.00E+06 | 0 | 0 | 0 | 0 | 0 | 0 | 0 |
| 5 | | 2.00E+06 | 0 | 0 | 0 | 0 | 0 | 0 | 0 |
| 5 | | 4.00E+06 | 0 | 0 | 0 | 0 | 0 | 0 | 0 |

Table S10: Parameters used in Figure 5F

| Bead  Number | Density  (beads/mL) | | Lower  Whisker | Lower Quartile | Median | Upper  Quartile | Upper  Whisker | Mean | SD |
| --- | --- | --- | --- | --- | --- | --- | --- | --- | --- |
| 0 | | 1.00E+06 | 66 | 70 | 74 | 75 | 78 | 72.6 | 4.66904701 |
| 0 | | 2.00E+06 | 14 | 15 | 17 | 20 | 28 | 17.8 | 5.6302753 |
| 0 | | 4.00E+06 | 15 | 16 | 16 | 21 | 22 | 18 | 3.24037035 |
| 1 | | 1.00E+06 | 21 | 25 | 26 | 29 | 34 | 27 | 4.84767986 |
| 1 | | 2.00E+06 | 72 | 76 | 80 | 80 | 83 | 79.2 | 4.2661458 |
| 1 | | 4.00E+06 | 44 | 48 | 53 | 58 | 65 | 53.6 | 8.2643814 |
| 2 | | 1.00E+06 | 0 | 0 | 0 | 1 | 1 | 0.4 | 0.54772256 |
| 2 | | 2.00E+06 | 0 | 3 | 3 | 4 | 5 | 3 | 1.87082869 |
| 2 | | 4.00E+06 | 18 | 23 | 26 | 28 | 31 | 25.2 | 4.96990946 |
| 3 | | 1.00E+06 | 0 | 0 | 0 | 0 | 0 | 0 | 0 |
| 3 | | 2.00E+06 | 0 | 0 | 0 | 0 | 0 | 0 | 0 |
| 3 | | 4.00E+06 | 2 | 3 | 3 | 3 | 5 | 3.2 | 1.09544512 |
| 4 | | 1.00E+06 | 0 | 0 | 0 | 0 | 0 | 0 | 0 |
| 4 | | 2.00E+06 | 0 | 0 | 0 | 0 | 0 | 0 | 0 |
| 4 | | 4.00E+06 | 0 | 0 | 0 | 0 | 0 | 0 | 0 |
| 5 | | 1.00E+06 | 0 | 0 | 0 | 0 | 0 | 0 | 0 |
| 5 | | 2.00E+06 | 0 | 0 | 0 | 0 | 0 | 0 | 0 |
| 5 | | 4.00E+06 | 0 | 0 | 0 | 0 | 0 | 0 | 0 |

Table S11: Parameters used in Figure 7A(ii)

| Cell  Number | Lower  Whisker | Lower Quartile | Median | Upper  Quartile | Upper  Whisker | Mean | SD |
| --- | --- | --- | --- | --- | --- | --- | --- |
| 0 | 59 | 64 | 64 | 78 | 81 | 69.2 | 9.67987603 |
| 1 | 19 | 22 | 36 | 36 | 38 | 30.2 | 8.95544527 |
| 2 | 0 | 0 | 0 | 0 | 3 | 0.6 | 1.34164079 |
| 3 | 0 | 0 | 0 | 0 | 0 | 0 | 0 |
| 4 | 0 | 0 | 0 | 0 | 0 | 0 | 0 |
| 5 | 0 | 0 | 0 | 0 | 0 | 0 | 0 |

Table S12: Parameters used in Figure 7B(ii)

| Cell  Number | Lower  Whisker | Lower Quartile | Median | Upper  Quartile | Upper  Whisker | Mean | SD |
| --- | --- | --- | --- | --- | --- | --- | --- |
| 0 | 17 | 17 | 19 | 20 | 23 | 19.2 | 2.48997992 |
| 1 | 67 | 72 | 72 | 75 | 75 | 72.2 | 3.27108545 |
| 2 | 7 | 7 | 8 | 8 | 8 | 7.6 | 0.54772256 |
| 3 | 0 | 1 | 1 | 1 | 2 | 1 | 0.70710678 |
| 4 | 0 | 0 | 0 | 0 | 0 | 0 | 0 |
| 5 | 0 | 0 | 0 | 0 | 0 | 0 | 0 |

Table S13: Parameters used in Figure 7C(iii)

| Cell  Number | Density  (cells/mL) | | Lower  Whisker | Lower Quartile | Median | Upper  Quartile | Upper  Whisker | Mean | SD |
| --- | --- | --- | --- | --- | --- | --- | --- | --- | --- |
| 0 | | 1.00E+06 | 72 | 75 | 75 | 78 | 81 | 76.2 | 3.42052628 |
| 0 | | 2.00E+06 | 17 | 17 | 19 | 20 | 23 | 19.2 | 2.48997992 |
| 0 | | 4.00E+06 | 14 | 15 | 15 | 17 | 21 | 16.4 | 2.79284801 |
| 1 | | 1.00E+06 | 17 | 21 | 23 | 23 | 27 | 22.2 | 3.63318042 |
| 1 | | 2.00E+06 | 67 | 72 | 72 | 75 | 75 | 72.2 | 3.27108545 |
| 1 | | 4.00E+06 | 37 | 38 | 41 | 42 | 46 | 40.8 | 3.56370594 |
| 2 | | 1.00E+06 | 1 | 1 | 2 | 2 | 2 | 1.6 | 0.54772256 |
| 2 | | 2.00E+06 | 7 | 7 | 8 | 8 | 8 | 7.6 | 0.54772256 |
| 2 | | 4.00E+06 | 17 | 30 | 30 | 31 | 34 | 28.4 | 6.58027355 |
| 3 | | 1.00E+06 | 0 | 0 | 0 | 0 | 0 | 0 | 0 |
| 3 | | 2.00E+06 | 0 | 1 | 1 | 1 | 2 | 1 | 0.70710678 |
| 3 | | 4.00E+06 | 7 | 11 | 12 | 14 | 15 | 11.8 | 3.1144823 |
| 4 | | 1.00E+06 | 0 | 0 | 0 | 0 | 0 | 0 | 0 |
| 4 | | 2.00E+06 | 0 | 0 | 0 | 0 | 0 | 0 | 0 |
| 4 | | 4.00E+06 | 0 | 2 | 2 | 3 | 5 | 2.4 | 1.81659021 |
| 5 | | 1.00E+06 | 0 | 0 | 0 | 0 | 0 | 0 | 0 |
| 5 | | 2.00E+06 | 0 | 0 | 0 | 0 | 0 | 0 | 0 |
| 5 | | 4.00E+06 | 0 | 0 | 0 | 0 | 1 | 0.2 | 0.4472136 |

Table S14: Parameters used in Figure S3.1

| Bead  Number | Beads (μm) | Lower  Whisker | Lower Quartile | Median | Upper  Quartile | Upper  Whisker | Mean | SD |
| --- | --- | --- | --- | --- | --- | --- | --- | --- |
| 0 | 10 | 11 | 11 | 11 | 14 | 16 | 12.6 | 2.30217289 |
| 0 | 15 | 14 | 15 | 17 | 20 | 28 | 17.8 | 5.6302753 |
| 1 | 10 | 81 | 83 | 87 | 87 | 88 | 85.2 | 3.03315018 |
| 1 | 15 | 72 | 76 | 80 | 80 | 83 | 79.2 | 4.2661458 |
| 2 | 10 | 1 | 2 | 2 | 3 | 3 | 2.2 | 0.83666003 |
| 2 | 15 | 0 | 3 | 3 | 4 | 5 | 3 | 1.87082869 |
| 3 | 10 | 0 | 0 | 0 | 0 | 0 | 0 | 0 |
| 3 | 15 | 0 | 0 | 0 | 0 | 0 | 0 | 0 |
| 4 | 10 | 0 | 0 | 0 | 0 | 0 | 0 | 0 |
| 4 | 15 | 0 | 0 | 0 | 0 | 0 | 0 | 0 |
| 5 | 10 | 0 | 0 | 0 | 0 | 0 | 0 | 0 |
| 5 | 15 | 0 | 0 | 0 | 0 | 0 | 0 | 0 |

Table S15: Parameters used in Figure S3.2

| Cell  Number | Cell Type | Lower  Whisker | Lower Quartile | Median | Upper  Quartile | Upper  Whisker | Mean | SD |
| --- | --- | --- | --- | --- | --- | --- | --- | --- |
| 0 | MKN-45 | 14 | 20 | 20 | 25 | 26 | 21 | 4.79583152 |
| 0 | MDA-MB-231 | 17 | 17 | 19 | 20 | 23 | 19.2 | 2.48997992 |
| 1 | MKN-45 | 65 | 66 | 72 | 72 | 75 | 70 | 4.30116263 |
| 1 | MDA-MB-231 | 67 | 72 | 72 | 75 | 75 | 72.2 | 3.27108545 |
| 2 | MKN-45 | 2 | 4 | 7 | 9 | 9 | 6.2 | 3.1144823 |
| 2 | MDA-MB-231 | 7 | 7 | 8 | 8 | 8 | 7.6 | 0.54772256 |
| 3 | MKN-45 | 1 | 1 | 1 | 5 | 5 | 2.6 | 2.19089023 |
| 3 | MDA-MB-231 | 0 | 1 | 1 | 1 | 2 | 1 | 0.70710678 |
| 4 | MKN-45 | 0 | 0 | 0 | 0 | 1 | 0.2 | 0.4472136 |
| 4 | MDA-MB-231 | 0 | 0 | 0 | 0 | 0 | 0 | 0 |
| 5 | MKN-45 | 0 | 0 | 0 | 0 | 0 | 0 | 0 |
| 5 | MDA-MB-231 | 0 | 0 | 0 | 0 | 0 | 0 | 0 |
